# Effect of a cheese rich in angiotensin-converting enzyme-inhibiting peptides (Gamalost^®^) and a Gouda-type cheese on blood pressure: results of a randomised trial

**DOI:** 10.3402/fnr.v60.32017

**Published:** 2016-08-04

**Authors:** Rita Nilsen, Are H. Pripp, Arne T. Høstmark, Anna Haug, Siv Skeie

**Affiliations:** 1 Department of Chemistry, Biotechnology and Food Science, Norwegian University of Life Sciences, Ås, Norway; 2 Oslo Centre of Biostatistics and Epidemiology, Research Support Services, Oslo University Hospital, Oslo, Norway; 3 Institute of Health and Society, University of Oslo, Oslo, Norway; 4 Department of Animal and Aquacultural Sciences, Norwegian University of Life Sciences, Ås, Norway

**Keywords:** bioactive peptides, intervention, dairy, Gamalost, Gouda, cardiovascular diseases

## Abstract

**Background:**

High blood pressure (BP) is the leading risk factor for global disease burden, contributing to 7% of global disability adjusted life years. Angiotensin converting enzyme (ACE)-inhibiting bioactive peptides have the potential to reduce BP in humans. These peptides have been identified in many dairy products and have been associated with significant reductions in BP.

**Objective:**

The objective of this trial was to examine whether a cheese rich in ACE-inhibiting peptides (Gamalost^®^), or a standard Gouda-type cheese could lower BP.

**Design:**

A total of 153 healthy participants were randomised to one of three parallel arms: Gamalost^®^ (*n=*53, 50 g/day for 8 weeks), Gouda-type cheese (*n=*50, 80 g/day for 8 weeks), and control (*n=*50). BP and anthropometric measurements were taken at the baseline and at the end, with an additional BP measurement midway. Based on BP at baseline, participants were categorised as having optimal BP (<120/<80 mmHg), normal-high BP (120–139/80–89 mmHg), or being hypertensive (>140/>90 mmHg). Questionnaires about lifestyle, health, and dietary habits were completed at baseline, midway and end.

**Results:**

In total, 148 participants (mean age 43, 52% female) completed the intervention. There were no differences among the three groups in relevant baseline characteristics. BP was reduced in the entire study population, but the cheese groups did not differ from control. However, in a subgroup of participants with slightly elevated BP, BP at 4 weeks of intervention seemed to be borderline significantly more reduced in the Gamalost^®^ group compared with the control group (Dunnett test: diastolic BP −3.5 mmHg, 95% confidence interval (CI) −7.3, 0.4, systolic BP: −4.3 mmHg, 95% CI −9.8, 1.1).

**Conclusion:**

An intention-to-treat analysis of the data showed no cheese effect upon BP compared to control, but Gamalost^®^ seemed to have a small, non-significant lowering effect on diastolic BP after 4 weeks in people with a normal-high BP.

Cardiovascular diseases (CVD) are the most common contributors to worldwide morbidity and mortality (1), and ischemic heart disease is the leading cause of death in the world (2). Hypertension is a major risk factor for CVD, and it has been estimated from prospective observational studies that just a 5 mmHg reduction in diastolic blood pressure (BP) would reduce the risk of stroke by 34% (3). BP was identified as the leading risk factor contributing to global disease burden in ‘the Global Burden of Disease Study 2010’, and it was estimated that 16.5% of all deaths can be attributed to high BP (4).

Hypertension is mostly treated pharmacologically, but lifestyle and dietary changes, such as weight loss and reduced salt intake, have been effective in preventing hypertension (5). The dietary approach to stop hypertension (DASH diet), which emphasises a high intake of dairy and fruits and vegetables, is one of the trials showing that diet is a successful tool to reduce hypertension (6). Dairy products are rich sources of protein, calcium, and potassium, which have all been shown to independently reduce BP (7–9). Dairy proteins are also one of the main sources of bioactive peptides in the human diet (10), which are present in varying amounts in different cheeses. These bioactive peptides have several known activities, including angiotensin-converting enzyme (ACE) inhibition. The function of ACE is to activate angiotensin II, a vasoconstrictor, as well as inactivating bradykinin, a vasodilator (11), resulting in increased BP. ACE-inhibiting peptides have been identified in several cheeses and other fermented milk products, including cheddar (12), manchego (13), asiago (14), and the traditional Norwegian cheese Gamalost^®^ (15). Some randomised, controlled trials have shown that fermented milks and extracts of ACE-inhibiting peptides from milk products can reduce BP in humans. A meta-analysis showed that food-derived peptides, such as the two lactotripeptdies valine-proline-proline (VPP) and isoleucine-proline-proline (IPP), had the potential to lower systolic BP by 5 mmHg (16).

Gamalost^®^ is a cheese made from skimmed milk that does not contain salt and is naturally low in fat (<1%) and very rich in protein (50%). A detailed account of the production of Gamalost^®^ has been described previously (15). The cheese is rich in bioactive peptides and was found to have a better ACE-inhibitory activity, in terms of the concentration of cheese peptides needed to inhibit 50% of ACE, than other cheeses (15, 17, 18). Compared to cheeses from other studies, Gamalost was one of the cheeses with the highest ACE-inhibitory potential (19). A cross-sectional study on Gamalost^®^ and BP was carried out in 2012 and showed that Gamalost^®^ intake frequency was associated with slightly lower systolic BP (20). Given its bitter taste and crumbly texture, Gamalost^®^ is not widely consumed, while mild and versatile Gouda-type cheeses have the highest consumption in Norway. A simulated human gastrointestinal digestion trial showed that the moderate ACE-inhibitory activity of Gouda-type cheeses increased greatly after digestion (18), making the cheese an interesting addition to this trial. We are not aware of any previously published randomised, controlled trials specifically investigating the effect of cheese on BP.

The aim of this work was to investigate whether the consumption of cheese might lower BP during 8 weeks of intervention.

## Materials and methods

### Subjects

Participants were recruited through local newspapers, radio, and television, from the general population. The target population was persons with moderately high blood BP, who were not medicated. Males and females over 18 years of age who spoke Norwegian fluently were included. Exclusion criteria included pregnancy and the use of BP-lowering medications.

### Design

**Fig. 1:**
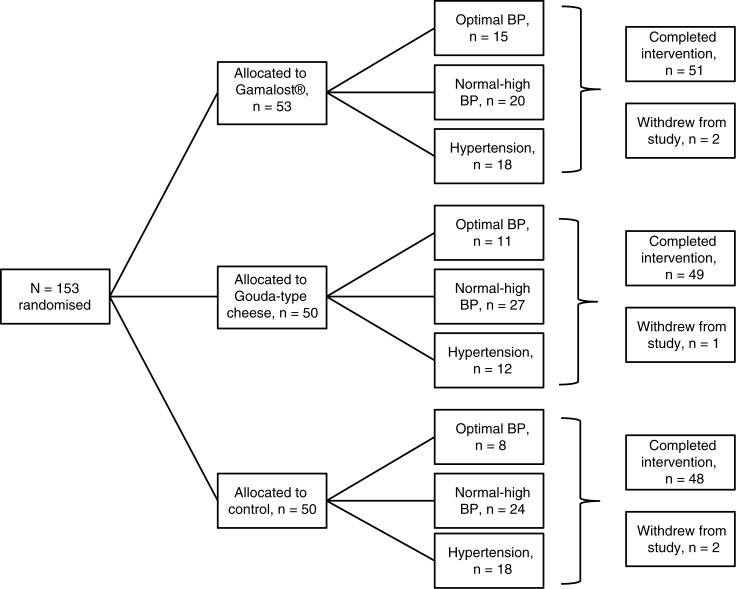
Flow chart of a single-blinded, randomised, controlled trial of Gamalost^®^ and Gouda-type cheeses and blood pressure in 153 Norwegian participants. BP, blood pressure.

The study performed was a randomised, single-blinded controlled trial with three parallel arms, as illustrated in Fig. 1. The intervention period lasted for 8 weeks with measurements taken at baseline, midway, and at the end of the trial. An independent person not involved in the study prepared the randomisation envelopes containing information on which intervention the participants would follow. Independent of baseline BP, the participants were handed envelopes by two independent persons not involved in the conduct of the study. The sample size estimate for a one-way analysis of variance with three groups, with a power of 0.80 and criterion for significance set at 0.05, yielded a sample size of 53 cases per group and a total of 159. We initially aimed for a larger sample of about 300 participants, but recruitment yielded a total sample size of 153.

This study was carried out at the Department of Chemistry, Biotechnology and Food Science, Norwegian University of Life Sciences, Aas, Norway, from April 2013 to July 2013 and was approved by the Regional Committees for Medical and Health Research Ethics (Oslo, Norway) on 7 March 2013 (2013/166) (registered at www.clinicaltrials.gov; NCT01913756). The study was conducted according to the guidelines laid down in the Declaration of Helsinki and written informed consent was obtained from all subjects.

### Interventions

Participants were randomised to one of three arms: Gamalost^®^, Gouda-type cheese, or control. All participants in the cheese groups were asked to maintain their habitual diet and not make any other major lifestyle changes. Subjects in the control group were asked to maintain their habitual diet, but to avoid the two intervention cheeses as well as similar cheeses. They were given lists of suggestions of other cheeses they could freely consume, such as blue cheese, mozzarella cheese, or cream cheese.

Participants in the Gamalost^®^ group were instructed to consume 50 g/day of the cheese, whereas participants in the Gouda-type cheese group were instructed to consume 80 g/day of the cheese. These amounts were chosen because they were judged to be higher than the average intake of each cheese, but not so high that the participants were unable to consume the designated amount. In order to have similar cheese protein intakes in the two cheese groups, the Gouda-type intervention was larger than the Gamalost^®^ intervention. The cheeses were not portioned, but the participants were provided with digital scales to accurately weigh out the daily intake. All the Gamalost^®^ cheeses were from the same batch and were ripened for 10 days. All the Gouda-type cheeses were from the same batch and were ripened for 3 months. The cheese needed for the duration of the trial was provided for all participants, and they were given more as needed. Furthermore, participants were instructed to freeze the Gamalost^®^ cheese throughout the trial so that the rapid proteolysis occurring in the cheese would not change the activity of the ACE-inhibiting peptides, as freezing does not affect the ACE-inhibitory activity of the cheese. The nutritional properties and ACE-inhibitory activity of the two cheeses can be found in Table 1. A portion of the study population filled out charts of weighed daily cheese intake, used to evaluate compliance with the cheese intake.

**Table 1:** Nutrient composition (per 100 g and daily intake) of intervention cheeses

	Gamalost^®^		Gouda-type cheese	
		
Nutrient	100 g	Daily intake, 50 g	100 g	Daily intake, 80 g
Energy^a^, kcal	213	107	351	281
Protein^a^, g	50	25	27	22
Fat^a^, g	1	0.5	27	22
Carbohydrates^a^, g	1	0.5	0	0
Calcium^a^, mg	160	80	800	640
Sodium^a^, mg	24	12	402	322
Magnesium^a^, mg	13	7	33	26
Potassium^a^, mg	98	49	77	62
IC_50_ ACE-inhibition^b^	0.34	0.34	0.59	0.59
ACE-inhibitory potential^c^, mg	0.24	0.12	0.03	0.02

a From TINE SA, manufacturer of the two cheeses

b IC_50_ per unit weight of freeze-dried pH 4.6 soluble fraction (SF), expressed as mg pH 4.6 SF per ml. From Qureshi et al. (15)

c ACE-inhibitory potential, expressed as mg captopril equivalents per cheese weight. From Qureshi et al. (15).

### Socio-demographic and dietary assessment

A questionnaire was developed for a cross-sectional trial on Gamalost^®^ intake and BP in a Norwegian population (20), and it was based on the previously validated questionnaire from the Oslo Health Study (main questionnaire and second supplementary questionnaire 1 of the Oslo Health Study). Experience from this cross-sectional trial showed that the questionnaire was suitable, but a couple more questions on food intake were added. The questionnaire contained questions on age, education, health, leisure time physical activity, medication, and supplement use, as well as several questions on diet, including some focusing specifically on dairy product intake. A translated version of the questionnaire used at baseline can be found in Supplementary file 1. Total dairy product intake was calculated by summarising the frequency of intake of all cheese, all milk, and fermented milk products. A revised version of the questionnaire containing only questions on food intake was distributed at the midway measurements, whereas a third version, which included some of the questions on health and physical activity from the first questionnaire, was used at the end of the trial. The second and third questionnaires were used to assess whether any major changes to diet and physical activity pattern occurred during the intervention period. Participants were also asked to record any difficulties they had with compliance. The last questionnaire also included a question regarding discomforts the participants may have experienced during the intervention period.

### BP measurements

BP was measured according to the American Heart Association recommendations (21). Participants rested for approximately 10 min before the measurement was taken using a Microlife BP A200 sphygmomanometer (Microlife, Widnau, Switzerland). In a sitting position, three consecutive measurements were taken and the average of the second and third measurements was used for analysis (automatically calculated by the BP device). If needed, the device took four measurements to get a more accurate reading. All BP measurements were taken between 06:30 and 10:30, after an overnight fast. Participants were notified of their BP and whether the BP was within the normal range or not. Based on baseline BP, participants were grouped into categories according to the guidelines published by the European Society of Hypertension and the European Society of Cardiology (22). Consequently, participants were categorised as optimal if systolic BP was <120 mmHg and diastolic BP was <80 mmHg, normal-high if systolic BP was 120–139 mmHg and/or diastolic BP was 80–89 mmHg, and hypertensive if systolic BP was ≥140 mmHg and/or diastolic BP was ≥ 90 mmHg.

### Anthropometric measurements

Height was measured to the nearest 0.1 cm using a portable stadiometer (Seca 217, Seca, Hamburg, Germany). Body weight was measured to the nearest 0.1 kg, without shoes or heavy clothing, using digital scales (TBF-300A Body Composition Analyzer, Tanita, Tokyo, Japan). Body mass index (BMI) was computed as weight (kg) divided by the square of height (m). Waist circumference was measured to the nearest 0.1 cm using a measuring tape (Seca 201 Circumference measuring tape, Seca), in accordance with World Health Organization recommendations, i.e. at the midpoint between the iliac crest and the lowest rib margin (23). All anthropometric measurements were also performed between 06:30 and 10:30.

### Statistical analyses

Prior to statistical analysis, the dataset was recoded by an independent person to remove information on intervention groups, hence the primary researchers were blinded while performing the analyses. Statistical analyses were performed according to the intention-to-treat principle. One-way ANOVA with Bonferroni correction for multiple comparisons or the chi-square (χ^2^) test were used as appropriate to assess differences between the intervention groups. Baseline characteristics are presented as mean (standard deviation) or percentages, where appropriate. The paired samples *t*-test was used to evaluate change in BP from start to end in each intervention group. Dunnett test was used to evaluate mean BP changes between each treatment group and the control group. A *p*<0.05 was considered statistically significant. All statistical analyses were performed using the SPSS 21.0 software package (IBM Corporation, Armonk, New York).

## Results

### Baseline characteristics

A total of 153 participants were included in the study from the beginning (*n*=53 in Gamalost^®^ group, *n*=50 each in both Gouda-type cheese and control groups). At baseline, 22% had optimal BP, 46% had moderately high BP, and 31% were hypertensive. Some baseline characteristics of the total study sample and the three groups are presented in Table 2. There were no major differences between the groups in selected health variables, salt, alcohol, or dairy product intake. BP was not significantly different at baseline, even though the prevalence of hypertension was slightly lower in the Gouda-type cheese group compared to the control group. The participants consumed on average seven servings of cheese per week, whereas Gamalost^®^ intake was predictably low with less than one serving per week.

**Table 2:** Baseline characteristics (mean (standard deviation (SD))) or %, by intervention group

	Intervention group								
		
	All (*n=*153)		Gamalost^®^ (*n=*53)		Gouda-type cheese (*n=*50)		Control (*n=*50)		
					
Characteristic	Mean	SD	Mean	SD	Mean	SD	Mean	SD	*p*
Gender, female (%)	52.3		50.9		60.0		46.0		0.4
Age (years)	43.1	16.4	41.2	17.0	42.7	15.8	45.5	16.4	0.4
Weight (kg)	77.2	14.8	75.6	13.7	76.0	13.6	79.9	16.8	0.3
Height (cm)	173.9	8.9	174.9	8.7	171.9	8.7	174.7	9.4	0.2
BMI (kg/m^2^)	25.7	3.7	24.6	3.3	25.6	3.5	26.0	3.7	0.1
Waist circumference (cm)	83.1	11.8	80.9	11.3	82.8	10.9	85.8	12.9	0.1
Systolic BP (mmHg)	132.3	17.2	131.5	19.3	130.6	14.7	134.8	17.2	0.4
Diastolic BP (mmHg)	82.4	9.8	82.5	10.6	81.4	8.9	83.1	10.0	0.7
Hypertension^a^ (%)	31.4		34.0		24.0		36.0		0.4
Education (years)	16.6	2.9	16.6	2.7	17.0	2.3	16.4	3.5	0.6
Smoking^b^ (%)	3.3		3.8		2.0		4.1		0.9
Physical activity^c^ (%)	38.4		39.6		36.7		38.8		0.9
Salt usage^d^ (%)	72.5		71.7		72.0		74.0		0.9
Alcohol consumption^e^ (%)	57.2		64.2		53.1		54.0		0.1
Total dairy^f^	18.4	11.9	19.7	12.9	17.5	10.1	18.0	12.7	0.6
Gouda-type cheese^f^	5.7	4.3	6.1	4.6	5.4	3.6	5.6	4.8	0.7
Gamalost^®^ f	0.7	1.9	0.6	1.7	0.6	1.8	0.8	2.2	0.9

a Percentage who have either SBP>140, or DBP>90

b percentage daily smokers

c percentage who reported moderate to hard physical activity more than 4 h per week

d percentage who salt their food

e percentage who consume alcohol > 1/week

f servings per week.

### BP changes

**Fig. 2:**
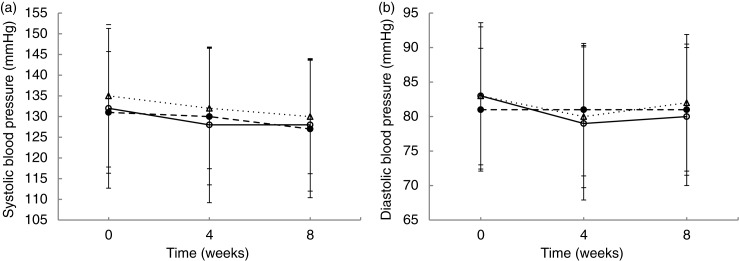
Mean values (standard deviation) for systolic (a) and diastolic (b) blood pressure at baseline, midway, and end in the three groups. ○Gamalost^®^, ●Gouda-type cheese, ΔControl.

Paired samples *t*-test (Supplementary file 2) showed that both systolic and diastolic BP decreased significantly from baseline to midway, and from baseline to the end of the trial in the entire study population. All groups had significant decreases in systolic BP (Fig. 2a, Supplementary file 2), whereas, at the end of the trial, only the Gamalost^®^ group had significantly decreased diastolic BP (Fig. 2b, Supplementary file 2). An intention-to-treat analysis of BP change, and comparing the intervention groups with the control group, showed no effect of the cheeses on midway or end BP (Supplementary file 3).

**Fig. 3:**
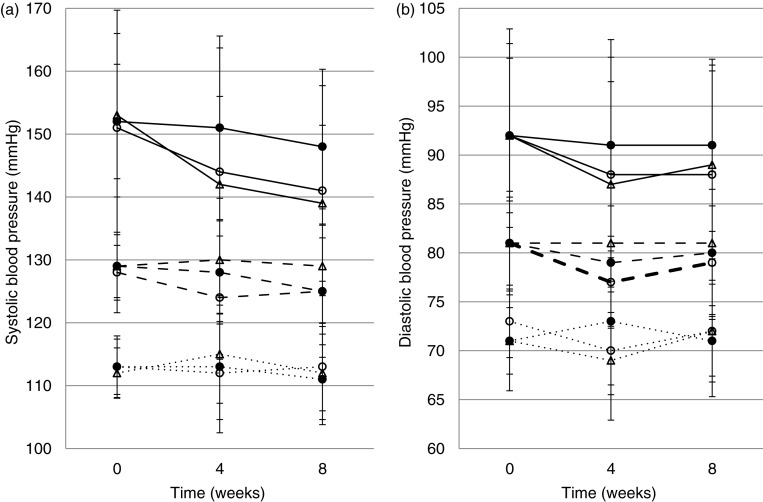
Systolic (a) and diastolic (b) blood pressure (mean (SD)) at baseline, midway, and end, in the three intervention groups. Solid lines: participants with hypertension at baseline; dashed lines: participants with normal-high BP at baseline, and dotted lines: participants with optimal BP at baseline. ○Gamalost^®^, ●Gouda-type cheese, ΔControl.

When stratifying by baseline BP category, the paired samples *t*-test (Supplementary file 4) showed that the participants with optimal BP at baseline did not have any reductions throughout the trial, as illustrated in Fig. 3. Figure 3a and b shows mean systolic and diastolic BP changes through the trial, respectively, grouped by BP category at baseline. Systolic BP decreased significantly in the hypertensive subgroup in both the Gamalost^®^ (*p=*0.001) and control (*p<*0.001) groups at both midway and end measurements. Systolic BP was also significantly lower at the end of the trial (*p=*0.049) for participants with normal-high BP in the Gouda-type cheese group. At the end of the trial, diastolic BP was significantly decreased in the normal-high BP (*p=*0.038) and hypertensive (*p=*0.004) subgroups of the Gamalost^®^ intervention group only.

Further analyses stratified by BP categories are presented in Table 3. Here, the intervention cheese groups are compared against the control group. Systolic BP shows a borderline significant effect of consuming Gamalost^®^ compared to control also in the normal-high BP subgroup (−4.3 mmHg, 95% CI −9.8, 1.1, *p=*0.14). Even though BP decreased overall in all groups, when comparing change in BP in those with hypertension at baseline, the Gouda-type cheese group had significantly higher midway and end systolic BP compared to the control group (midway: 10.5 mmHg, 95% CI 0.9, 20.2, *p=*0.03, end: 10.1 mmHg, 95% CI 1.6, 18.6, *p=*0.02). In the Gamalost^®^ group, there seems to be a small borderline significant effect on diastolic BP compared to control at week 4, for participants with normal-high BP at baseline (−3.5 mmHg, 95% CI −7.3, 0.4, *p=*0.08). At 8 weeks, the association remained but was less significant.

**Table 3:** Stratified analysis based on blood pressure category at baseline, comparing control group with the two cheese diets

		4 weeks (midway)					8 weeks (end)			
			
		Baseline BP	Mean BP	BP change	Difference from control (95% CI)	*p*	Mean BP	BP change	Difference from control (95% CI)	*p*
Systolic BP										
Hypertensive	Control	152.7 (3.1)	141.8 (3.3)	−10.9 (2.2)			139.1 (2.9)	−13.7 (2.5)		
	Gamalost^®^	150.8 (4.4)	144.2 (4.6)	−6.7 (2.8)	4.3 (−4.3, 12.9)	0.43	141.3 (3.9)	−9.50 (2.5)	4.2 (−3.4, 11.8)	0.36
	Gouda-type	151.5 (2.6)	147.9 (3.5)	−0.4 (3.7)	10.5 (0.9, 20.2)	0.03	147.9 (3.5)	−3.6 (2.1)	10.1 (1.6, 18.6)	0.02
Normal-high	Control	129.2 (2.1)	129.7 (2.1)	0.3 (1.7)			128.7 (1.9)	−0.6 (1.7)		
	Gamalost^®^	128.4 (1.4)	124.4 (2.2)	−4.1 (1.7)	−4.3 (−9.8, 1.1)	0.14	125.4 (1.9)	−3.0 (1.7)	−2.4 (−8.1, 3.4)	0.55
	Gouda-type	128.6 (1.0)	127.9 (1.6)	−0.5 (1.5)	−0.8 (−6.0, 4.3)	0.91	124.9 (2.1)	−3.5 (1.7)	−2.9 (−8.3, 2.5)	0.37
Optimal	Control	111.5 (1.4)	114.9 (2.8)	3.4 (2.4)			111.8 (2.6)	0.3 (2.2)		
	Gamalost^®^	112.5 (1.3)	112.3 (2.5)	−0.1 (1.9)	−3.5 (−11.7, 4.7)	0.50	113.5 (2.0)	0.5 (1.6)	0.2 (−6.8, 6.4)	1.0
	Gouda-type	112.9 (1.3)	127.9 (1.6)	−0.4 (3.1)	−3.8 (−12.5, 5.0)	0.50	109.7 (2.3)	−3.5 (1.9)	−3.8 (−10.2, 2.7)	0.31
										
Diastolic BP										
Hypertensive	Control	91.9 (2.2)	141.8 (3.3)	−5.1 (2.0)			88.5 (2.3)	−3.4 (2.1)		
	Gamalost^®^	92.2 (2.6)	144.2 (4.6)	−4.1 (1.4)	1.0 (−4.5, 6.5)	0.89	87.6 (2.6)	−4.6 (1.4)	−1.2 (−6.8, 4.4)	0.85
	Gouda-type	92.0 (2.3)	147.9 (3.5)	−0.9 (2.1)	4.1 (−2.0, 10.3)	0.23	92.3 (2.5)	−0.8 (1.8)	2.6 (−3.6, 8.9)	0.54
Normal-high	Control	80.8 (1.0)	80.5 (1.6)	−0.1 (1.3)			80.6 (1.5)	0.1 (1.5)		
	Gamalost^®^	81.0 (1.2)	77.5 (1.1)	−3.6 (1.1)	−3.5 (−7.3, 0.4)	0.08	78.8 (1.3)	−2.2 (1.0)	−2.2 (−6.4, 2.1)	0.42
	Gouda-type	80.9 (0.8)	79.3 (1.1)	−1.4 (1.0)	−1.3 (−4.9, 2.4)	0.65	79.7 (1.3)	−0.9 (1.3)	−0.9 (−4.9, 3.1)	0.84
Optimal	Control	70.5 (1.2)	68.5 (1.2)	−2.0 (1.3)			71.8 (2.4)	1.3 (1.8)		
	Gamalost^®^	73.0 (1.0)	69.7 (1.9)	−3.7 (1.7)	−1.7 (−7.7, 4.3)	0.72	71.9 (1.4)	−1.5 (1.5)	−2.8 (−8.0, 2.4)	0.36
	Gouda-type	71.3 (1.5)	79.3 (1.1)	1.3 (2.0)	3.3 (−3.1, 9.7)	0.38	71.2 (1.1)	−0.8 (1.3)	−2.1 (−7.5, 3.4)	0.59

Values are mean (SE), 2-sided *p*-values for the difference from control (Dunnett test).

### Other changes

At the end of the intervention, waist circumference decreased significantly in all three intervention groups (*p<*0.001), whereas weight was only significantly reduced in the Gamalost^®^ and control groups (data not shown). Dairy product and Gamalost^®^ intake was significantly different between the groups at the end of the trial (ANOVA, data not shown, *p<*0.001), with total dairy intake increasing in the two cheese groups and decreasing in the control group. Five participants failed to complete the trial, as illustrated in Fig. 1, due to adverse events unrelated to the study itself.

## Discussion

This randomised, controlled trial showed systolic BP reductions in all intervention groups, and reductions in diastolic BP in the Gamalost^®^ group after 8 weeks of intervention. However, compared to the control, there was no BP-lowering effect of Gamalost^®^, a cheese rich in ACE-inhibiting peptides, or of a standard Gouda-type cheese in 153 subjects recruited from a general healthy population. When participants were stratified by baseline BP, there was a non-significant effect of consuming Gamalost^®^ compared to control at 4 weeks in participants with normal-high BP, but this was not present at 8 weeks.

In this trial, systolic BP was significantly reduced in hypertensive subjects in both the Gamalost^®^ and control groups, but not the Gouda-type group. It is uncertain why BP was reduced in the control group, but it could be a result of the statistical phenomenon known as regression to the mean (24). Another reason for the reduction of systolic BP in the control group could be the seasonal variations in BP that are known to occur throughout the year (25). BP tends to be higher in the winter months and lower in the summer, which is consistent with the start of this study in April and ending in June. Even though there was no significant difference in baseline BP between the three groups, the Gouda-type cheese group had fewer subjects with hypertension compared to the Gamalost^®^ and control groups. This could possibly explain why the hypertensive subgroup in the Gouda-type cheese group did not have the same reductions in BP as the other two groups. It was hypothesised that during the intervention, BP would decrease in the Gamalost^®^ group compared to the control, due to the high intake of ACE-inhibiting bioactive peptides. A borderline significant reduction in diastolic BP in the Gamalost^®^ group was seen in the subcategory of subjects with normal-high BP. The association was less significant at 8 weeks, which could be explained by regression to the mean or problems with compliance. This borderline significant change in diastolic BP is in accordance with a larger diet intervention trial on the effect of the Mediterranean diet on BP, which found a small significant effect on diastolic but not systolic BP (26). In the previous cross-sectional trial (20), the opposite occurred as systolic BP, but not diastolic BP, was significantly lower with higher intakes of Gamalost^®^. With the high prevalence of high BP and hypertension in the Norwegian and worldwide population, a small lowering of mean BP in the normal-high BP subgroup in the Gamalost^®^ group compared to control at 4 weeks could be clinically meaningful if they were able to achieve a significant effect beyond 4 weeks. The normal-high BP category was the largest subgroup in all three intervention groups, leading to the assumption that results from these groups have the best statistical power. In the general population, this subgroup of people is also those who might benefit from lifestyle changes, for example, a diet including a cheese rich in ACE-inhibiting peptides, such as Gamalost^®^.

It has been estimated that a small reduction in diastolic BP of 2 mmHg could reduce the risk of coronary heart disease by 6% (27), indicating that only small reductions are needed for cheese and dairy products to have a clinically meaningful effect on BP. Previous randomised, controlled trials on the BP-lowering effect on milk-derived bioactive peptides showed mixed results. VPP and IPP, derived from casein, are usually considered the two lactotripeptides with the most promising antihypertensive potential (28). However, in a double-blinded placebo-controlled trial on subjects with elevated BP (SBP ≥ 140 mmHg) given concentrates of these two peptides, they did not exert any BP-lowering effect compared to the placebo group (28). A similar trial using a milk fermented with *Lactobacillus helveticus*, which gave a product naturally rich in VPP and IPP, found a significant BP-lowering effect on diastolic BP, which was not maintained after 4 weeks’ intervention in the normal-high BP category, whereas both systolic and diastolic BP was decreased in the hypertensive category until 4 weeks (29). Many trials investigating the BP-lowering effect of foods have used extracts of foods or synthetic foods/drinks containing active ingredients that may occur naturally in foods. Trials that use actual foods as the intervention, such as the current trial, have mixed results in terms of BP reductions. A BP-lowering effect of foods consumed as part of a normal diet in a free living population has been observed for foods such as kiwifruit (male smokers) (30), flaxseed (peripheral artery disease patients) (31), and fermented milk (buttermilk) (moderately hypercholesterolemic subjects) (32), whereas no significant effects were found for other foods, such as walnuts (33).

Information obtained from participants in the cross-sectional trial (20), which included many people who regularly consumed Gamalost^®^, indicated that 50 grams of Gamalost^®^ was a feasible daily intake of the cheese. It is possible that a true BP-lowering effect of Gamalost^®^ would be observed if the serving was increased, but we judged that a bigger serving would be unlikely to be tolerated by regular and non-consumers. The study population in the current intervention trial was recruited from the general population and they had no underlying conditions and diseases. We were unable to recruit solely subjects with increased BP at baseline, as evidenced by the initial BP in the overall study population (132/82 mmHg). A significant correlation between initial BP and the change in BP has been found (34), with initial higher BP showing greater response to the BP-lowering agent, suggesting that, should further trials on the BP-lowering effect of Gamalost^®^ be performed, subjects with optimal BP should be excluded. With the inclusion of normotensive participants in the trial, it is possible that this study is actually underpowered since we would not expect a reduction in BP in those with normal BP. Even though many cheeses have been found to be rich in ACE-inhibiting bioactive peptides, the results from the present trial suggest that the effect may not be transferrable to healthy human populations through the amount of cheese consumed in this trial. A meta-analysis of randomised, controlled trials on the effect of VPP and IPP on BP found that the response to treatment was generally greater in Japanese studies compared to European studies (35). The authors suggested that one cause was the typically higher habitual intake of dairy products in Europe, which may have an effect on the BP response to lactotripeptides treatment. The fact that participants in the control group in the current trial could consume dairy products could have confounded the results of this trial and contributed towards the null findings. Suggestions for future trials are to include a wash-out period before the baseline, during which participants do not consume dairy products, and to remove dairy products from the diet of the control group.

It is uncertain why waist circumference was reduced in all groups from baseline to the end of the trial. The questionnaire used in this trial was not detailed enough to detect changes in energy intake, but it did not seem that food intake changed significantly. The change in waist circumference could be caused by increased physical activity as the season changed from late winter/early spring to late spring/early summer. It is also possible that the reduction in waist circumference was due to the phenomenon known as the Hawthorne effect, in which participants in a trial will change their behaviour because they know they are being observed.

A limitation of this trial is the use of a sphygmomanometer for in-office BP measurements, as opposed to ambulatory 24-h BP measurements, which would produce values of higher accuracy. BP varies throughout the day and the current trial is therefore unable to distinguish whether the participants had an effect of the intervention on nocturnal BP. White-coat hypertension, reported to occur in about 15–35% of people (21, 36, 37), is a source of error that could be greatly reduced by ambulatory BP measurements (38). If the baseline BP measurements were falsely high, it is expected that BP will decrease slightly on subsequent visits, a result of getting used to the situation and regression to the mean. However, subjects were randomly allocated to groups and statistical analyses were adjusted according to the subject's baseline BP. At the baseline BP measurement, the participants were informed of their BP. They were not given any medical advice, but they were told if their BP was outside of the recommended range, they could make an appointment with their general practitioner. None of the participants reported starting any medical antihypertensive treatments during the trial. Furthermore, since the participants were of generally good health and had, on average, a BP within the normal range at baseline, the generalisability of these findings to populations with a higher BP may be somewhat limited. The participants were only provided with the intervention cheese, and though they were asked to maintain their habitual diet, we had only partial control of the diet during the trial.

The strengths of this study include the relatively long duration and design of the trial, specifically that it is a randomised, single-blinded controlled trial. The retention of participants in the trial was good, with only five subjects lost to follow-up, and the same number of subjects dropped out in the Gamalost^®^ group and the control group.

In conclusion, when compared to the mean change in BP in the control group, there was no major effect of a cheese rich in ACE-inhibiting peptides or a standard Gouda-type cheese on BP in a general population. However, when stratified by BP category at baseline, there was a non-significant reduction in diastolic BP in the group that consumed the cheese rich in ACE-inhibiting peptides, compared to the control group of those participants with normal-high BP at 4 weeks of intervention. The current results suggest cheeses rich in ACE-inhibiting bioactive peptides may not have an effect on BP when consumed in moderate amounts, but further similar trials on other cheeses should be performed to evaluate these findings.

## Supplementary Material

Effect of a cheese rich in angiotensin-converting enzyme-inhibiting peptides (Gamalost^®^) and a Gouda-type cheese on blood pressure: results of a randomised trial
