# Supplementary material for: Effect of a cheese rich in angiotensin-converting enzyme-inhibiting peptides (Gamalost®) and a Gouda-type cheese on blood pressure: results of a randomised trial
Source: Food Nutr Res. 2016 Aug 4;60:10.3402/fnr.v60.32017. doi: 10.3402/fnr.v60.32017 (PMC4976624; doi:10.3402/fnr.v60.32017)
Supplement: Effect of a cheese rich in angiotensin-converting enzyme-inhibiting peptides (Gamalost®) and a Gouda-type cheese on blood pressure: results of a randomised trial [file FNR-60-32017-s001.docx]

**Supplementary Material**

Supplementary Material 1. Questionnaire at baseline

1. Your health

1.1 How would you describe your present state of health? (Check only one answer)

| Poor | Not very good | Good | Very good |
| --- | --- | --- | --- |
|  |  |  |  |

1.2 Do you have any of these illnesses, or have you suffered from of them in the past?

|  | Yes | No | Age on first occasion |
| --- | --- | --- | --- |
| Asthma |  |  |  |
| Chronic bronchitis/emphysema |  |  |  |
| Diabetes |  |  |  |
| Osteoporosis |  |  |  |
| Myocardial infarction |  |  |  |
| Angina pectoris (cardiac spasm) |  |  |  |
| Stroke/cerebral haemorrhage (“drip”) |  |  |  |
| High blood pressure |  |  |  |

2. Where you grew up/where you live

2.1 Where did you live for most of the time before you reached the age of 16 years?

(Check one alternative and specify)

| Same place |  |
| --- | --- |
| Another county in Norway | County: |
| ­Outside Norway | Country: |

2.2 Have you moved in the course of the last five years?

­ (Check only one answer)

| No | Yes, once | Yes, several times |
| --- | --- | --- |
|  |  |  |

3. Weight

|  | kg |
| --- | --- |

3.1 Assess your weight when you were 25 years old: ­­­

4. Food and drinks

4.1 How often do you usually eat the following kinds of foods?

(Check the appropriate answer on each line)

|  | Seldom/Never | 1-3 times/month | 1-3 times/week | 4-6 times/week | 1-2 times/day | >3 times/ day |
| --- | --- | --- | --- | --- | --- | --- |
| Fruit/berries­ ­ ­ ­ |  |  |  |  |  |  |
| Cheese (all kinds)­ ­ ­ |  |  |  |  |  |  |
| Potatoes­ ­ |  |  |  |  |  |  |
| Vegetables­ ­ ­ |  |  |  |  |  |  |
| Fatty fish (e.g. salmon, trout, mackerel, herring) |  |  |  |  |  |  |
| Gouda-type cheese |  |  |  |  |  |  |
| Brown whey cheese |  |  |  |  |  |  |
| Gamalost |  |  |  |  |  |  |
| Liver paté |  |  |  |  |  |  |
| Salami |  |  |  |  |  |  |
| Ham |  |  |  |  |  |  |
| Cured ham |  |  |  |  |  |  |
| Mackerel in tomato |  |  |  |  |  |  |
| Jam |  |  |  |  |  |  |
| Caviar |  |  |  |  |  |  |
| Mayonnaise-based sandwich salads |  |  |  |  |  |  |

4.2 To what degree have you changed your intake of the foods in 4.1 the last 3 months?

| Not at all | Some | A lot |
| --- | --- | --- |
|  |  |  |

4.3 Do you eat some of the foods in 4.1 periodically?

| Yes | No |
| --- | --- |
|  |  |

If yes, which ones have you eaten a lot the last month?

|  |
| --- |
|  |

4.4 What kind of fat do you use most often? (Check only one on each line)

|  | Butter | Hard margarine | Soft/light margarine | Oils | Do not use |
| --- | --- | --- | --- | --- | --- |
| On bread |  |  |  |  |  |
| For cooking |  |  |  |  |  |

4.5 Do you take the following food supplements? (Check only one on each line)

|  | Yes, daily | Sometimes | No |
| --- | --- | --- | --- |
| Cod liver oil, cod liver oil capsules, fish oil capsules |  |  |  |
| Vitamin and/or mineral supplements |  |  |  |

4.6 How much do you usually drink of the following?

(Check one per line).

|  | Seldom/never | 1-6 glasses/week | 1 glass/ day | 2-3 glasses/day | >4 glasses/day |
| --- | --- | --- | --- | --- | --- |
| Whole milk, yoghurt |  |  |  |  |  |
| Kefir ­ ­ ­ ­ ­ |  |  |  |  |  |
| Semi-skimmed milk, low fat yoghurt ­ ­ ­ ­ ­ |  |  |  |  |  |
| Cultura, Biola |  |  |  |  |  |
| Skimmed milk (sour/sweet) ­ ­ ­ ­ ­ |  |  |  |  |  |
| Fruit juice ­ ­ ­ ­ ­ |  |  |  |  |  |
| Water ­ ­ ­ ­ ­ |  |  |  |  |  |
| Cola drinks ­ ­ ­ ­ ­ |  |  |  |  |  |
| Other fizzy drinks/thirst quenchers |  |  |  |  |  |

4.7 Do you usually drink fizzy drinks / cola?

| With sugar | Without sugar |
| --- | --- |
|  |  |

4.8 To what degree have you changed your intake of fizzy drinks in the last 3 months?

| Not at all | Some | A lot |
| --- | --- | --- |
|  |  |  |

4.9 How many cups of coffee or tea do you drink daily?

(Write 0 if you do not drink coffee or tea daily)

| Number cups coffee |  |
| --- | --- |
| Number cups tea |  |

­­ ­­4.10 Do you normally salt your food?

| Yes, a lot | Yes, some | No |
| --- | --- | --- |
|  |  |  |

4.11 How often have you consumed alcohol in the course of the past year?

(Low alcohol beer and non-alcoholic beer are not included)

| 4-7 times/week | 2-3 times/week | Once/week | 2-3 times/month | Once/month | Few times/year | None past year | Never had alcohol |
| --- | --- | --- | --- | --- | --- | --- | --- |
|  |  |  |  |  |  |  |  |

­

4.12 When you drink, do you usually drink: (Check more than one if applicable)

| ­­Beer | Wine | Spirits |
| --- | --- | --- |
|  |  |  |

4.13 To what degree have you changed your intake of alcohol in the last 3 months?

| Not at all | Some | A lot |
| --- | --- | --- |
|  |  |  |

4.14 If you have changed your diet the last three months, how has it changed? (Check one or more)

| Less fat |  |  |
| --- | --- | --- |
| More fat |  |  |
| Less carbohydrates |  |  |
| More carbohydrates |  |  |
| More fruit and vegetables |  |  |
| Less salt |  |  |
| More fatty fish |  |  |
| I have not made any changes |  |  |
| Other |  | |

5. Tobacco

5.1 Have you smoked/do you smoke daily?

| Yes, currently | Yes, previously | Never |
| --- | --- | --- |
|  |  |  |

5.2 If you smoke daily now, or have smoked before

| How many cigarettes do you or did you usually smoke daily? |  |
| --- | --- |
| How many years altogether have you smoked? |  |

5.3 Have you used snus/do you use it daily?

| Yes, currently | Yes, previously | Never |
| --- | --- | --- |
|  |  |  |

5.4 If you use snus daily now, or have used it previously:

| How many snus do you or did you usually use daily? |  |
| --- | --- |
| How many years altogether have you used snus? |  |

6. Education and work

­­6.1 How many years of schooling/education have you completed altogether? ­

|  | Years |
| --- | --- |

6.2 What is your highest achieved education? (Check only one)

| Primary and secondary school |  |
| --- | --- |
| Upper secondary school |  |
| College, 1 year |  |
| College/university, 3 years (Bachelor) |  |
| College/university, 5 years (Master) |  |
| College/university, > 5 years |  |
| PhD |  |

6.3 Are you currently employed?

| Yes, full time | Yes, part time | No | Student |
| --- | --- | --- | --- |
|  |  |  |  |

7. Physical activity

7.1 What kind of physical activity have you undertaken in you spare time in the course of the past year?

Estimate a weekly average for the year. From home to work is regarded as spare time. Answer both questions.

|  | Hours per week | | | |
| --- | --- | --- | --- | --- |
|  | None | Less than 1 | 1-2 | 3 or more |
| Light exercise  You do not sweat or feel out of breath |  |  |  |  |
| Hard physical activity  You sweat and feel out of breath |  |  |  |  |

7.2 Describe the extent of movement and bodily exertion in your spare time. If the activity varies considerably, e.g. between summer and winter, then give an average. The question applies to the past year only.

(Check the appropriate answer)

| Read, watch TV or other sedentary activity? |  |
| --- | --- |
| Walk, cycle or move about in some other way at least 4 times/week  (This should include walking or cycling to work, Sunday stroll/walk, etc.) |  |
| Take part in physical exercise/sport, do heavy gardening work?  (Note that the activity must take place at least 4 times a week) |  |
| Exercise hard or take part in competitive sport regularly and several times a week |  |

8. Use of medicines

8.1 Do you take any of these medicines?

|  | Currently | Earlier | Never |
| --- | --- | --- | --- |
| Medicine for high blood pressure |  |  |  |
| Cholesterol-reducing medicine |  |  |  |

8.2 If you have used any of the medicines in 8.1 in the last 4 weeks, give the name and reason for using them:

| How long have you used this medicine? | |  |  |
| --- | --- | --- | --- |
| Name of medicine | Reason for use | Up to 1 year | More than 1 year |
|  |  |  |  |

9. Questions for women

9.1 Are you currently pregnant?

| Yes | No | Not sure | Past fertile age |
| --- | --- | --- | --- |
|  |  |  |  |

9.2 If you use the p-pill, mini-pill, p-injection, hormone loop or oestrogen;

which preparation do you use?

|  |
| --- |

10. Other

| Gender |  |  |
| --- | --- | --- |
| Age |  |  |
| To be filled out by health personnel at inclusion in the trial (week 1) | | |
| Height |  |  |
| Weight |  |  |
| Waist circumference |  |  |
| Blood pressure |  |  |
| Blood glucose |  |  |
| Blood sample ID |  |  |
| ID number |  |  |

Supplementary Material 2. Mean difference (MD^1^) with 95% CI in blood pressure in each group, paired samples t-test comparing start to 4 and 8 weeks follow-up.

|  |  | Study population | | |  | Gamalost^®^ | | |  | Gouda-type cheese | | |  | Control | | |
| --- | --- | --- | --- | --- | --- | --- | --- | --- | --- | --- | --- | --- | --- | --- | --- | --- |
|  |  | MD | 95% CI | *P* |  | MD | 95% CI | *P* |  | MD | 95% CI | *P* |  | MD | 95% CI | *P* |
| Systolic BP^2^, mmHg | Change to 4 weeks | -2.7 | -4.2, -1.1 | 0.001 |  | -3.9 | -6.5, -1.3 | 0.004 |  | -0.5 | -3.2, 2.3 | 0.727 |  | -3.5 | -6.5, -0.5 | 0.023 |
|  | Change to 8 weeks | -4.4 | -6.0, -2.9 | <0.001 |  | -4.4 | -7.0, -1.8 | 0.001 |  | -3.5 | -5.8, -1.3 | 0.003 |  | -5.4 | -8.5, -2.2 | 0.001 |
|  |  |  |  |  |  |  |  |  |  |  |  |  |  |  |  |  |
| Diastolic BP, mmHg | Change to 4 weeks | -2.3 | -3.3, -1.3 | <0.001 |  | -3.8 | -5.3, -2.2 | <0.001 |  | -0.7 | -2.4, 1.0 | 0.421 |  | -2.3 | -4.4, -0.3 | 0.029 |
|  | Change to 8 weeks | -1.6 | -2.7, -0.6 | 0.002 |  | -2.9 | -4.4, -1.4 | <0.001 |  | -0.9 | -2.7, 0.9 | 0.319 |  | -1.1 | -3.3, 1.1 | 0.333 |

^1^MD, mean difference

^2^BP, blood pressure

Supplementary Material 3. Blood pressure changes during intervention, comparing control group with the two cheese diets. Values are mean (SE), 2-sided *P*-values for the difference from control (Dunnett test)

|  |  | 4 weeks (midway) | | | |  | 8 weeks (end) | | | |
| --- | --- | --- | --- | --- | --- | --- | --- | --- | --- | --- |
|  | Baseline BP | Mean BP | BP change | Difference from control (95% CI) | *P* |  | Mean BP | BP change | Difference from control (95% CI) | *P* |
| Systolic BP^1^ |  |  |  |  |  |  |  |  |  |  |
| Control | 134.8 (2.4) | 131.8 (2.1) | -3.5 (1.5) |  |  |  | 129.8 (2.0) | -5.4 (1.6) |  |  |
| Gamalost^®^ | 131.5 (2.7) | 128.0 (2.6) | -3.9 (1.3) | -0.4 (-4.8, 3.9) | 0.97 |  | 128.0 (2.2) | -4.4 (1.3) | 1.0 (-3.2, 5.2) | 0.83 |
| Gouda-type | 130.6 (2.1) | 130.5 (2.4) | -0.5 (1.4) | 3.0 (-1.4, 7.4) | 0.23 |  | 127.4 (2.4) | -3.5 (1.1) | 1.8 (-2.4, 6.1) | 0.53 |
|  |  |  |  |  |  |  |  |  |  |  |
| Diastolic BP |  |  |  |  |  |  |  |  |  |  |
| Control | 83.1 (1.4) | 80.9 (1.5) | -2.3 (1.0) |  |  |  | 82.1 (1.4) | -1.1 (1.1) |  |  |
| Gamalost^®^ | 82.5 (1.5) | 79.1 (1.5) | -3.8 (0.8) | -1.5 (-4.2, 1.3) | 0.40 |  | 80.2 (1.4) | -2.9 (0.7) | -1.8 (-4.6, 1.1) | 0.28 |
| Gouda-type | 81.4 (1.3) | 80.9 (1.4) | -0.7 (0.8) | 1.6 (-1.2, 4.5) | 0.33 |  | 80.7 (1.4) | -0.9 (0.9) | 0.2 (-2.7, 3.1) | 0.98 |

^1^BP, blood pressure

Supplementary Material 4. Mean change in blood pressure in each group, stratified by baseline blood pressure, paired samples t-test for change from start to end.

|  |  |  | Gamalost | | Gouda-type cheese | | Control | | Study population | |
| --- | --- | --- | --- | --- | --- | --- | --- | --- | --- | --- |
|  |  |  | Mean | *P* | Mean | *P* | Mean | *P* | Mean | *P* |
| Systolic BP^1^ | Hypertension | Baseline | 151 |  | 152 |  | 153 |  | 152 |  |
|  |  | Change midway | -6.67 | 0.030 | -0.42 | 0.913 | -10.94 | <0.001 | -6.71 | <0.001 |
|  |  | Change end | -9.50 | 0.001 | -3.58 | 0.120 | -13.67 | <0.001 | -9.58 | <0.001 |
|  |  | n | 18 |  | 12 |  | 18 |  | 48 |  |
|  | Normal high | Baseline | 128 |  | 129 |  | 129 |  | 129 |  |
|  |  | Change midway | -4.05 | 0.026 | -0.54 | 0.727 | 0.29 | 0.871 | -1.33 | 0.170 |
|  |  | Change end | -3.00 | 0.091 | -3.54 | 0.049 | -0.64 | 0.716 | -2.44 | 0.016 |
|  |  | n | 20 |  | 26 |  | 22 |  | 68 |  |
|  | Optimal | Baseline | 113 |  | 113 |  | 112 |  | 112 |  |
|  |  | Change midway | -0.14 | 0.941 | -0.40 | 0.900 | 3.38 | 0.208 | 0.66 | 0.642 |
|  |  | Change end | 0.46 | 0.771 | -3.50 | 0.101 | 0.25 | 0.913 | -0.87 | 0.425 |
|  |  | n | 13 |  | 10 |  | 8 |  | 32 |  |
| Diastolic BP | Hypertension | Baseline | 92 |  | 92 |  | 92 |  | 92 |  |
|  |  | Change midway | -4.06 | 0.008 | -0.92 | 0.671 | -5.06 | 0.020 | -3.65 | 0.001 |
|  |  | Change end | -4.56 | 0.004 | -0.75 | 0.691 | -3.39 | 0.130 | -3.17 | 0.004 |
|  |  | n | 18 |  | 12 |  | 18 |  | 48 |  |
|  | Normal high | Baseline | 81 |  | 81 |  | 81 |  | 81 |  |
|  |  | Change midway | -3.55 | 0.006 | -1.35 | 0.186 | -0.10 | 0.944 | -1.61 | 0.020 |
|  |  | Change end | -2.20 | 0.038 | -0.92 | 0.470 | -0.05 | 0.976 | -1.01 | 0.173 |
|  |  | n | 20 |  | 26 |  | 22 |  | 68 |  |
|  | Optimal | Baseline | 73 |  | 71 |  | 71 |  | 72 |  |
|  |  | Change midway | -3.71 | 0.051 | 1.30 | 0.526 | -2.00 | 0.178 | -1.72 | 0.119 |
|  |  | Change end | -1.54 | 0.336 | -0.80 | 0.550 | 1.25 | 0.510 | -0.58 | 0.519 |
|  |  | n | 13 |  | 10 |  | 8 |  | 32 |  |

^1^BP, Blood pressure
